# Do independent treatment centers offer more value than general hospitals? The case of cataract care

**DOI:** 10.1111/1475-6773.13201

**Published:** 2019-08-20

**Authors:** Florien M. Kruse, Stef Groenewoud, Femke Atsma, Onno P. van der Galiën, Eddy M. M. Adang, Patrick P. T. Jeurissen

**Affiliations:** ^1^ Scientific Center for Quality of Healthcare (IQ healthcare) Radboud Institute for health Sciences Nijmegen The Netherlands; ^2^ Zilveren Kruis Leusden The Netherlands; ^3^ Department of Health Evidence Radboud University Medical Center Nijmegen The Netherlands

**Keywords:** efficiency, health care costs, independent treatment centers, quality of care, value

## Abstract

**Objective:**

To identify differences between independent treatment centers (ITCs) and general hospitals (GHs) regarding costs, quality of care, and efficiency.

**Data Sources:**

Anonymous claims data (2013‐2015) were used. We also obtained quality indicators from a semipublic platform.

**Study Design:**

This study uses a comparative multilevel analysis, controlling for case mix, to evaluate the performance of ITCs and GHs for patients diagnosed with cataract.

**Data Collection:**

Reimbursement claims were extracted from existing claims databases of the largest Dutch health insurer. Quality indicators were obtained by external agencies through a mixed‐mode survey.

**Principal Findings:**

There are no stark differences in complexity of cases for cataract care. ITCs seem to perform surgeries more frequently per care pathway, but conduct a lower number of health care activities per surgical claim. Total average costs are lower in ITCs compared with GHs, but when adjusted for case mix, the differences in costs are lower. The findings with the adjusted quality differences suggest that ITCs outperform GHs on patient satisfaction, but patients’ outcomes are similar.

**Conclusion:**

This finding supports the postulation—based on the focus factory theory—that ITCs can provide more value for cataract care than GHs.

## 1 INTRODUCTION

Provoked by the increasing pressure to sustain rising health care costs, policy makers are seeking for more efficient ways to organize health care delivery. To pursue the vision of a value‐based health care system (measuring outcomes, costs, and creating integrated practice units), some scholars proposed to utilize more specialized services instead of full‐service providers.^1^ The theoretical assumption behind this argues that specialization provides the right conditions to improve efficiency and quality of care. Reallocating (ambulatory) elective care from full‐service hospitals (ie, academic and general hospitals) to independent treatment centers (ITCs) could be one important response to improve efficiency within the broader health care system.

ITCs are usually smaller independent providers which generally focus on one patient group, specialism, or treatment.^2^ In several health care systems, ITCs are more profit‐oriented than general hospitals (GHs).^3^ ITCs seem to embody to a greater extent the theoretical concept of the focus factory.^4^, ^5^, ^6^ This theory postulates that harmonizing the care portfolio and specialization would lead to better performance due to repetition, experience, and homogeneity of tasks. The aim would be to enhance the expertise of the health care provider and improve efficiency. These efficiency gains could then lower operational costs,^2^, ^7^ through standardization and by reallocating expertise and equipment to just one place. Subsequently, reductions in overhead costs could be made possible. Furthermore, quality could be improved by means of routine and cultivating from continued learning. In line with Michael Porter's theory of Value‐Based Health Care (VBHC) which defines value as patient‐related outcomes relative to costs,^1^ ITCs would theoretically achieve more value for the same procedure compared with full‐service hospitals.

In many countries, the number of ITCs has risen steeply over the past decades. This increase is partly due to technological advances: more treatments can be reallocated to ambulatory care settings. Also, policy makers became more receptive toward ITCs, since many health care systems opted for a more market‐driven system. In the United States, the number of Medicare‐certified ITCs (called independent Ambulatory Surgery Centers in the US) doubled between 1991 and 2001 (1460‐3371), but recently this growth has slowed down.^8^, ^9^ In the UK, the number of ITCs peaked in the mid‐90s and has been declining since,^10^ however, the total spending on the ITC sector increased with 39 percent between 2013/14 and 2016/17.^11^ In the Netherlands, the number of ITC concerns has been growing steadily from 81 in 2008 to 241 in 2015.^6^ ITCs started to emerge in the Netherlands when an act (1998) came into force allowing ITCs to provide reimbursable medical care for a limited array of treatments. That act was introduced in order to reduce waiting lists and to gain control over the for‐profit clinics.^12^, ^13^ The formal distinction between ITCs and hospitals was abolished with the introduction of the Health Care Institutions Admission Act in 2005, which regulates the approval of reimbursable care providers. Now, both hospitals and ITCs are formally defined as medical specialist care providers; however, ITCs are still different in practice and categorized as different types of entity, by, for instance, patients associations, health insurers, and ITCs themselves.

Some empirical evidence on the relative performance of ITCs exists, most of which comes from the United States. However, comparative studies scrutinizing cost and quality simultaneously are lacking. The studies that analyzed quality of care either find equivocal results^14^, ^15^ or find no clear medical quality advantages for ITCs over full‐service hospitals.^14^, ^16^ Studies covering patients’ experiences are also inconsistent, with one UK study finding no significant differences for the overall reported patients’ experiences,^17^ whereas one other study identified higher satisfaction rates among ITCs users compared with NHS facilities.^18^ In contrast, the independent sector in the UK charged higher prices than NHS hospitals (unclear whether this disparity still exists),^19^ and evidence points to the fact that ITCs in the UK are not always more efficient—only patients with hip or knee replacements had a shorter length of stay when treated in an ITC.^20^ At the same time, findings from both the UK and the US suggest that ITCs might be cherry‐picking and treat less‐complex patients compared with hospitals.^20^, ^21^, ^22^, ^23^, ^24^, ^25^

From the demand side, it seems that the characteristics of patients seeking care from ITCs differ from those of patients seeking care from hospitals. The independent sector in the UK historically serves the interests of private practices of NHS consultants and target a more affluent clientele with additional amenities and shorter waiting lists.^3^, ^20^ Also in the United States, patients who are not insured via Medicaid more often chose to visit an ITC.^25^ In the Netherlands, there is still a knowledge gap regarding the patterns of referral for patients visiting ITCs. One report from 2013 provides more insight on the motivations underlying patients’ choice of health care provider: Patients going to ITCs often make the choice themselves (43 percent)—fewer of whom make a choice themselves to opt for care in a GH (38 percent).^26^ Furthermore, not all ITCs are contracted by all health insurers, while GHs more often are contracted by all four major health insurers—with a contracting index of 0.53 for ITCs and 0.88 for hospitals.^27^ Therefore, people with an indemnity health care insurance package will probably be more inclined to opt for ITCs. These insurance packages cover ITC care even when the ITC has no contract from this respective health insurer. From this perspective, people who could afford a more indemnity health care insurance package—people with a higher socioeconomic status (SES)—might have better access to ITCs. However, a recent report found no relationship between income and the choice for those insurance coverage that limits the choice of health care providers.^28^ Another reason why there might be a certain selection of patients visiting ITCs is that, according to guidelines set by the Dutch health care inspectorate, ITCs should refrain from treating patients of ASA (American Society of Anesthesiologists) type III, which are patients with severe systemic diseases.^29^

This study focuses on cataract care, a care modality often provided by ITCs. Cataract care is a classical example that illustrates the shift from inpatient care toward ambulatory care settings: “Cataract surgery has dramatically evolved from a procedure done almost exclusively as a routine inpatient procedure with a hospital stay up to 1 week to an outpatient operation with minimal limitations on the patient's postoperative activity.”^30^ In the Netherlands, most cataract surgical procedures are now outpatient and ITCs play a substantial role in delivering them.^12^, ^31^ There is a growing need to optimize cataract care delivery due to aging societies which means that the demand for cataract surgery will increase.^32^

In 2006, the Netherlands implemented a number of market‐oriented reforms of the health care system and the ITC enterprises subsequently grew. The focus factory theory would predict that ITCs would provide better value, but there remains uncertainty as to whether ITCs really do outperform GHs. To the best of our knowledge, this is the first study that scrutinizes the performance of ITCs in the Dutch health care system and assesses the added value of ITCs. Cataract surgery provided by ITCs is compared with GHs over the period 2013 to 2015. Our aim was to provide insight into the case mix‐adjusted differences between ITCs and GHs regarding costs, quality, and efficiency.

## 2 METHODS

### 2.1 Data

Our data are based on (anonymous) insurer claims and cover the period 2013‐2015. We were able to include 4.5 million beneficiaries who were covered by the insurance company Achmea. This sample is highly representative: Achmea had a market share of 31.1 percent in 2015, making it the biggest health insurer in the Netherlands.^33^ Achmea has the highest market share across a wide geographic area in the Netherlands, whereas three other largest health insurers are more geographically concentrated. Achmea claims data therefore offer a good degree of geographical representativeness.^34^ Furthermore, the beneficiaries of the main health insurers reflect the diversity of the Dutch population, because the health insurers cross‐subsidize costs among the more loss‐making and more profit‐generating clientele.^35^ We extracted ophthalmological claims for people with a cataract diagnosis, based on the diagnosis code included in the claims data. All individual ophthalmological claims within a single year were obtained.

We use the annual cross‐sectional inclusion of claims per patient to define the patients’ care pathway. This means that all the ophthalmological claims that were claimed that year for one specific patient diagnosed with cataract were assigned to their patients’ care pathway. Patients who received care from multiple providers during their care pathway were excluded from analysis, constituting between 1.6 percent and 2.0 percent of the patients. Data on quality of care were obtained from a platform that collects quality measures for health insurers. This specific database is owned and managed by the national database for insurers (Vektis). The quality data were obtained by means of a mixed‐mode survey (not part of the current study), contracting two different external parties to manage the data collection. The national number of cataract surgeries per provider was attained from the same platform (Vektis). Data were linked through a unique identifier assigned by Vektis and are on concern level (a concern can have multiple locations). This unique identifier was also used to identify ITCs and GHs as the identifier codes are structured in such a way that the type of provider can be easily detected. Comparisons between GHs and ITCs are our main interest because academic and tertiary care hospitals deviate too much from the ITC organizational model, mainly because of their teaching objectives and their more complex patient base. Tertiary care and academic hospitals were categorized manually by means of the identifier codes. The descriptive statistics of these types can be found in the supplementary material.

### 2.2 Study variables

In the Netherlands, providers are paid through a diagnostic‐related groups system. Such groups are called “care products” (DRGs) and also include outpatient care.^36^ For the care products used in this study, the price per DRG is determined through bilateral negotiations between health insurers and providers. Volume encompasses the total number of DRGs claimed in one care pathway, which could, for example, be consultation and diagnostic DRGs. Two types of cataract surgical DRGs are included: complex and standard. The number of health care activities contained in one surgical reimbursed DRG serves as a measure for efficiency. The different health care activities are categorized into four categories. These four categories contain the following number of activities: 14 diagnostic, 5 anesthetic, 4 surgical, 2 consultation activities and 1 day care admission activity. A cataract surgical DRG has to contain one of the four surgical cataract activities. For example, one surgical DRG can contain one surgical activity, three different diagnostic activities (eg, biometric test, optical coherence tomography, and an electrocardiographic assessment), and two different consultations. The precise number of activities could only be analyzed for 2015 because before that year providers were not obliged to share this information with their health insurers.

The patient characteristics should determine the possible case mix differences. Besides age and gender, this includes the level of multimorbidity, ocular comorbidity, and SES.^37^ SES was derived from the postal codes of the patients, using the SES scores of 2014 provided by The Netherlands Institute for Social Research (Sociaal en Cultureel Planbureau SCP).^38^ This proxy is based on education, income, and position in the labor market of all the inhabitants within that neighborhood. Zero equals the average Dutch neighborhood, and minus zero indicates a lower than average SES neighborhood, whereas above zero indicates a higher SES than average. To assess possible multimorbidity, we grouped pharmaceutical claims of patients and used those as a proxy to identify whether they have one of the 27 chronic conditions included in the Dutch risk‐adjusted contribution classification system. A patient was classified as multimorbid if they had two or more chronic conditions. We included ocular comorbidity as a separate confounder variable, since ocular comorbidity can have an impact on possible complications after cataract surgery.^39^ To measure this, we used a proxy: diabetic type I and II and glaucoma—also obtained from the pharmaceutical claims. The models that include quality were adjusted for total surgical volume, accounting for the volume‐quality relationship.^40^ This includes the total number of cataract surgical claims per provider, so not solely the claims filled by Achmea.

Finally, we used patient‐reported data from the Dutch Consumer Quality Index Cataract Questionnaire (CQI Cataract) to assess for quality of cataract surgery.^41^ The quality indicators are the Net Promoter Score (NPS) and a patient‐reported outcome measure (PROM). The NPS is a common management tool to measure patient satisfaction and asks the opinion of patients on “How likely is it that you would recommend this hospital or clinic for a cataract operation to a friend or colleague?”. The ratio of the number of promoters over the number of detractors makes up the NPS. The PROM used for this study measures the perceived outcome of patients 4 weeks after their cataract surgery based on 12 different questions which measures the patient‐reported outcome after surgery. For instance, if the patient, 4 weeks after the cataract operation, can see better at short distance (all PROM questions are available in the supplementary material). Both NPS and PROM were available on the level of the individual providers. We have NPS and PROM data for 2013 and 2014. However, for both years two different PROM scales were used: For 2013, a 4‐point ordinal scale was used; for 2014, this was a 5‐point scale, which makes comparisons between these two years troublesome.

### 2.3 Statistical analysis

Our descriptive statistics outline the unadjusted interprovider differences regarding the characteristics of cataract patients (ie, case mix), type of surgical procedures, volume, price, and total costs. A mixed‐model approach is used to analyze the association between type of provider (ITCs vs GHs) and the dependent variables: number of health care activities (2015), total costs of claims (2013‐2015), and the quality parameters NPS and PROM (2013‐2014). In the models, we accounted for clustering of patients within hospitals, including a random intercept for provider level, and adjusted for confounders such as case mix differences. Actual claims costs are skewed to the right; therefore, the total claims costs were logarithmically transformed in the multilevel model. The multilevel models are tested for better fit with the nontransformed cost models utilizing the Akaike information criterion (AIC).^42^ The GHs are used as our reference category.

The mixed model with the number of health activities as dependent variable controls for (a) SES; (b) multimorbidity; (c) gender; (d) aged 85 or older; and (e) ocular comorbidity. The model, which includes the total log costs as dependent variable, controls for the same case mix confounders mentioned above and additionally controls for (a) conservative treatment; (b) the number of surgical procedures; and (c) complex cataract surgery. The model which incorporates both costs and quality restricts to patients who at least had one surgical cataract procedure. Total costs are used as a control when quality is the dependent variable and vice versa. This last model builds upon the last mentioned model, but adds total volume of the provider as control variable. The volume estimator has three categories (ie, low volume, middle, and high volume providers) and is based on the figures of how volume is distributed: the lower 25 percent (≤700), middle (>700‐<3000), and upper 25 percent (≥3000) of 2013 and 2014.

## 3 RESULTS

### 3.1 Patient characteristics

Table 1 shows the characteristics of the sample of patients per type of provider (2015). The dataset includes 29 cataract ITCs. In total, this dataset contains around 50 000 patients who received cataract care (including academic and tertiary hospitals). In 2013, ITCs had 19.3 percent share of cataract patients (in supplementary material), which further grew to 24.1 percent in 2015. The type of treatment provided to patients is relatively similar between ITCs and GHs: Around 56 percent of the cataract patients received standard cataract surgery; 6.5 percent received complex cataract surgery; and 38 percent received no surgery.

**Table 1:** Descriptive statistics: provider characteristics, type of treatments, patient characteristics, volume, price, and total costs (2015)

		Cataract	
		ITCs	GHs
*Provider characteristics*			
Total number of providers	N	29	52
Number of patients	N	11 526	20 901
	%	24.11	43.72
*Type of treatment*			
Standard cataract surgery	%	55.69	55.92
Complex cataract surgery	%	6.55	5.14
No surgery	%	37.75	38.91
*Patient characteristics*			
Average age	Mean	72.26 (9.77)	73.20 (10.10)
<18 y	%	0.08	0.26
>85 y	%	8.32	10.29
Men	%	41.07	42.75
Average number of chronic conditions	Mean	2.15 (1.65)	2.24 (1.72)
Average number of Diabetes I patients	Mean	0.05 (0.22)	0.08 (0.27)
Average number of Diabetes II patients	Mean	0.14 (0.35)	0.18 (0.39)
Average number of Glaucoma patients	Mean	0.30 (0.46)	0.12 (0.33)
SES	Mean	−0.06 (1.20)	−0.28 (1.16)
*Volume*			
Number of DRGs per patient care pathway of cataract care	Mean	1.45 (0.63)	1.41 (0.63)
Number of cataracts per patients’ care pathway	Mean	0.91 (0.81)	0.84 (0.77)
≥2 cataract per patients’ care pathway	%	28.42	22.96
*Price*			
Price DRG for standard cataract surgery	Mean	1009.22 (46.07)	1095.15 (110.51)
Price DRG for complex cataract surgery	Mean	1250.58 (114.99)	1391.07 (154.93)
*Total costs*			
Total costs for cataract—conservative	Mean	115.43 (58.31)	117.27 (65.41)
Total costs for patients with 1 cataract operation	Mean	1057.38 (109.38)	1151.20 (164.47)
Total costs for patients with 2 cataract operations	Mean	2085.43 (167.86)	2272.05 (287.40)

Note

Standard deviations in parentheses.

Patient characteristic statistics illustrate that there are small differences in the complexity of patients for cataract care between ITCs and GHs. The mean age is lower in ITCs. The percentage of patients who are 85 years or older is much lower in ITCs than GHs. The average number of chronic conditions illustrate that ITCs’ patients have less comorbidity, and the average number of patients with diabetes indicates possible lower ocular comorbidity. The average SES of patients going to ITCs is higher compared with GHs. Glaucoma is the only indicator that suggests that ITCs might be treating a more complex patient group since in ITCs the number of patients with glaucoma is higher compared with GHs. Glaucoma might have a negative impact on the postoperative visual acuity,^39^ but on the other hand, when glaucoma has been detected early enough, a higher share of glaucoma patients does not necessarily reflect the complexity of those treated, because with medication their symptoms can be successfully suppressed.^43^, ^44^ In conclusion, these findings indicate that overall complexity of ITC patients for cataract care do not differ strongly from GHs.

### 3.2 Volume

The number of DRGs and the number of surgical claims show that ITCs submit a slightly higher number of claims during a care pathway than GHs do (Table 1). Nevertheless, the average number of surgeries is higher within ITCs, with, on average, 0.91 cataract operations per care pathway, while GHs have an average of 0.84.

### 3.3 Price and total claims costs

The descriptive statistics on charged DRG prices and total claims costs of the care pathway are also exhibited in Table 1. The DRG prices, which the insurer negotiates with ITCs, are substantially lower for cataract surgery than prices for GHs: on average 85.9 euros less for standard cataract surgery and 140 euros for complex cataract surgery. For patients with one cataract operation, the total cost differences are on average 94 euros per care pathway, and for patients with two cataract operations, this gap widens to 187 euros—both accounting for approximately 8 percent in cost savings. When patients receive conservative treatment, there seem to be relatively small cost differences between ITCs and GHs. These descriptive findings are consistent over the years 2013 and 2014 (see Tables S1‐S4).

When adjusted for case mix, the total claims costs for cataract care in ITCs stay lower compared with GHs (Table 2). However, this difference becomes smaller, to a difference of 5 percent in 2015 (based on the exponentiated coefficient of −0.05 since the log costs are the outcome variable), compared with the unadjusted descriptive statistics of 8 percent. In addition, in 2013 ITCs seem to have been, in contrast to 2014 and 2015, actually slightly more expensive than GHs.

**Table 2:** Relationship between type of provider (ITCs vs GHs) and the log costs of all claims per patients’ care pathway (2013‐2015)

	2013 log costs	2014 log costs	2015 log costs
GHs	Reference	Reference	Reference
ITC	0.05*** (0.00)	−0.02*** (0.00)	−0.05*** (0.00)
Observations	47 931	47 176	47 396

Note

Controlled for academic hospitals, tertiary care hospitals, SES, gender, multimorbidity, ocular comorbidity, aged 85 or older, 2 or more operations, type of operation (conservative and complex).

Standard errors in parentheses.

*** *P* < .01.

### 3.4 Efficiency

Efficiency in this study is defined as the number of activities in a surgical claim, where fewer activities are perceived as more efficient. Results in Table 3 suggest that ITCs are more efficient in providing cataract surgery. ITCs carry out fewer health care activities within each surgical cataract DRG compared with GHs. The day care procedures (ie, a number of hours of nursing care spent within a nursing ward) are significantly shorter in ITCs. The number of anesthetic procedures also depicts a strong contrast: ITCs seem to do no anesthetic procedures. The explanation for ITCs reporting almost no anesthetic procedures is because there are no health care activities for anesthetic eye drops. (Anesthetic eye drops is a commonly used anesthetic for less‐complex patients.^45^) Only optometric therapy is a more frequent procedure among ITCs. This might well correspond with our reasoning that ITCs seem to be more efficient, since optometrists can serve as cheaper substitutes for ophthalmologists.^46^

**Table 3:** Descriptive statistics of the number of health care activities within the surgical cataract DRGs (2015)

		ITCs	GHs
Activities within complex cataract surgery	Total	4.27 (2.02)	5.48 (2.30)
	Diagnostic	0.82 (1.07)	1.04 (1.31)
	Anesthetics	0.00 (0.07)	0.51 (0.92)
	Day care	0.31 (0.47)	0.72 (0.48)
	Optometric consultation	0.44 (0.57)	0.26 (0.67)
Activities within standard cataract surgery	Total	4.14 (1.70)	4.56 (2.07)
	Diagnostic	0.78 (0.95)	0.86 (1.13)
	Anesthetics	0.01 (0.16)	0.38 (0.81)
	Day care	0.36 (0.48)	0.57 (0.52)
	Optometric consult	0.38 (0.56)	0.25 (0.59)

Note

Standard deviations in parentheses.

These differences between ITCs and GHs persist when adjusted for case mix (Table 4). The efficiency gained by ITCs seems to be higher with complex cataract surgical claims compared with standard cataract surgical claims. Approximately, and on average, (adjusted for case mix factors) ITCs perform 0.5 fewer activities compared with GHs; for a complex cataract surgical claim, this is approximately 1 activity fewer.

**Table 4:** Relationship between type of provider (ITCs vs GHs) and the number of health care activities within the two surgical claims (2015)

	For standard cataract surgical claim	For complex cataract surgical claim
GHs	Reference	Reference
ITCs	−0.42*** (0.03)	−1.19*** (0.10)
Observations	34 863	3 299

Note

Controlled for academic hospitals, tertiary care hospitals, SES, gender, multimorbidity, ocular comorbidity, gender, aged 85 or older.

Standard errors in parentheses.

*** *P* < .01.

### 3.5 Patient value

Table 5 illustrates that, when the model controls for quality, the claims costs in ITCs remain lower compared with GHs for both 2013 and 2014 with 7 percent (exp(−0.07) ≈ 0.93). This is higher than the model with the adjusted claims costs (Table 2; 5 percent difference), which does not control for quality differences, which means that ITCs perform better when quality of care is also taken into account. Quality differences between ITCs and GHs demonstrate that ITCs score significantly better on the NPS compared with GHs. However, the dissimilarity of the PROM scores is marginal and inconsistent. In other words, ITCs seem to perform better on patient satisfaction compared with GHs, but there are no differences in the patient‐reported outcomes after cataract surgery.

**Table 5:** Relationship between type of cataract care provider (ITCs vs GHs) and the log costs of all claims per patients’ care pathway and quality of care (NPS and PROM), rotating log costs and quality of care as outcome or control variable (2013 and 2014)

	Cataract	Cataract	Cataract	Cataract	Cataract	Cataract
	2013 log costs^a^	2014 log costs^a^	2013 NPS^b^	2014 NPS^b^	2013 PROM^b^	2014 PROM^b^
GHs	Reference	Reference	Reference	Reference	Reference	Reference
ITC	−0.07*** (0.00)	−0.07*** (0.00)	0.16*** (0.00)	0.13*** (0.00)	0.01*** (0.00)	−0.01*** (0.00)
Observations	29 486	28 582	29 486	28 582	29 486	28 582

Note

Controlled for academic hospitals, tertiary care hospitals, SES, gender, multimorbidity, ocular comorbidity, aged 85 or older, high and low volume providers, 2 or more operations, type of operation (complex).

Standard errors in parentheses.

a Controlled for NPS and PROM.

b Controlled for log costs.

*** *P* < .01.

## 4 DISCUSSION

Our results indicate that ITCs, compared with GHs, can be value‐adding entities for cataract care. This finding supports the “focus factory” thesis that typify ITCs. Total costs of cataract claims are lower for ITCs compared with GHs, although the adjusted cost differences are somewhat smaller than the unadjusted costs. Lower costs seem to be partly driven by lower negotiated prices, since ITCs tend to have a slightly higher number of claims per cataract care pathway. Our findings suggest that ITCs are able to offer those lower prices for cataract surgery, due to performing less health care activities within cataract surgical claims and through more intense use of optometrists. In addition, lower fixed costs (eg, lower overhead) and perhaps lower margins could be other reasons why cataract ITCs are able to offer lower prices. With respect to quality of care, the results are mixed. NPS scores are significantly higher for ITCs compared with GHs, while differences in the PROM scores are inconsistent and marginal. In other words, patients’ experiences are better in ITCs, but the differences in patient‐reported improvement after cataract surgery are opaque and do not seem to differ. Overall, these quality measures exhibit different results, underlying the need to measure the different quality dimensions.

We find limited selection of low‐severity patients for cataract surgery by ITCs, which is in line with the findings in Meyerhoefer et al,^22^ but goes against the studies that do find case mix differences.^20^, ^25^ Furthermore, this study also seems to support that different quality indicators can show contrasting results.^15^ A general trend that seems to emerge is that ITCs score better on patients’ satisfaction,^18^ but not on patient‐reported outcomes of the treatment.^14^

This study has some strengths. We were able to use claims data from a big sample of the Dutch population utilizing multiple years. Secondly, this study is one of the first that empirically studies the relative performance of the ITC market in a number of areas (ie, costs, quality and efficiency). Thirdly, this study takes a broader perspective of the patients’ care pathway, instead of only comparing surgical claims. Fourthly, we were able to separate claim reimbursements from actual activities, identifying process efficiency differences between ITCs and GHs.

Our study is also subject to some limitations. (a) The quality indicators used in this study were not optimal. Quality data for cataract surgery were on provider level, and not individual treatment level. In addition, the PROM scores were part of the CQI questionnaire, but were derived from the nonvalidated part. (b) The study design adjusts for relevant case mix differences; however, we cannot exclude the possibility that unobserved case mix differences influenced our results. Case mix differences can be a serious confounder because the referral patterns seem to differ between ITC and GH patients. Quasi‐experimental evaluation tools should be considered for future research, when longitudinal and/or more detailed data become available, to limit the unobserved variances between ITCs and GHs. This is especially relevant for treatments for which the outcomes are more case mix‐dependent (eg, total hip replacements). For instance, instrumental variable (IVs) models could be used for this purpose. These models should then take into account potential selection bias at two different levels (ie, provider and patient).^47^ (c) The proxy used in this study to measure patients’ care pathway is a relatively crude measure since it is based on the annual cross‐sectional claims. This study would ideally have used the patients’ care pathway identifier, which is included in the Dutch claims data, but due to serious irregularities, this identifier was deemed unreliable. (d) There is a risk that our proxy for efficiency—the number of health care activities—might not fully capture the differences in the resources used since this could vary by the different health care activities. (e) An additional limitation is that hospital may systematically cross‐subsidize their activities on more competitive markets such as cataract surgery. However, due to negotiated global budgets with the additional requirement to deliver additional services if patients need them, the actual room to cross‐subsidize has become more limited in recent years.

This study contributes to our limited understanding of the relative performance of ITCs compared with GHs. However, some important questions remain unanswered. The first question is whether the care provided by ITCs serves as a substitute for hospital care. US findings reveal that a growing penetration of ITCs does not necessarily induce a decline in ICT‐sensitive services in hospitals.^48^ The second question relates to the concern whether suppliers induce demand. Several studies from the United States have indicated that this is sometimes the case,^49^, ^50^ particularly among physician‐owned health care providers.^16^ Based on our own estimation, of the ITCs contracted for cataract care, 68 percent are physician‐owned. The phenomenon of supplier‐induced demand could very well affect the Dutch physician owners. They do need to maintain and improve the financial health of their organization because their incomes depend on it. However, in the Netherlands, the extent of these financial incentives is limited; not only does the Netherlands prohibit health care providers from allocating profits to owners or third parties but it also imposes a salary cap on Dutch physicians who are board members of ITCs. We note that the United States' prohibition on self‐referrals under the Stark Laws also tries to limit the issue of undesirable incentives. (This is a restriction which the Netherlands has not imposed.) Notwithstanding the regulation already in place, policy makers and health care purchasers should consider the possibilities of supplier‐induced demand when designing reimbursement legislation and contracting strategies. Thirdly, we have found that ITCs more often carry out two cataract operations per care pathway than GHs. It is beyond the scope of this study to assess whether this indicates that ITCs are undertaking unnecessary cataract operations but it does raise concerns which merits further investigation. Fourthly, this study cannot exclude the possibility of upcoding practices. Our findings do hint toward concerns of this sort. We observe an irregular combination of a higher number of DRGs and cataract operations per care pathway among ITCs, but a lower number of activities within each claim, while at the same time most case mix indicators indicate that ITCs are not treating a more complex patient base compared with GHs. Nevertheless, DRGs are automatically defined from the filled health care activities, and upcoding might be less plausible vs the idea that efficiency gains drive our finding.

The role of ITCs within future health care systems is still up for debate. Currently, GHs need to continue providing elective ambulatory care surgery if they are to ensure their long‐term financial survival; therefore, GHs will likely resist the reallocation of these services to ITCs. Moreover, increasing ITC penetration may increase the risks of efficiencies of scope driving out efficiencies of scale. In conclusion, for some elective surgeries ITCs could potentially enhance value of modern health care systems, but policy makers do need to be alert to possible adverse effects.
