# Supplementary material for: Do independent treatment centers offer more value than general hospitals? The case of cataract care
Source: Health Serv Res. 2019 Aug 20;54(6):1357–65. doi: 10.1111/1475-6773.13201 (PMC6863231; doi:10.1111/1475-6773.13201)
Supplement: Supplementary file 2 [file HESR-54-1357-s002.docx]

Supplementary Material: “Do Independent Treatment Centers offer more value than general hospitals? The case of cataract care.”

Table S1. Descriptive statistics tertiary and academic hospitals (2015)

|  | | Academic hospitals | Tertiary care hospitals |
| --- | --- | --- | --- |
| *Provider characteristics* | |  |  |
| Total number of providers | | 8 | 19 |
| Number of patients | N | 2108 | 13267 |
|  | % | 4.41 | 27.75 |
| *Type of treatment* |  |  |  |
| Standard cataract surgery | % | 43.93 | 53.8 |
| Complex cataract surgery | % | 11.67 | 7.25 |
| No surgery | % | 43.93 | 38.92 |
| *Patient characteristics* | |  |  |
| Average Age | mean | 66.19  (16.28) | 72.42 (10.11) |
| <18 years | % | 3.32 | 0.17 |
| >85 years | % | 6.07 | 9.14 |
| Men | % | 49.00 | 43.40 |
| Average number of pharmaceutical products | Mean | 2.30  (1.84) | 2.38  (1.75) |
| Average number of Diabetes I patients | Mean | 0.11  (0.32) | 0.09 (0.29) |
| Average number of Diabetes II patients | Mean | 0.16  (0.37) | 0.21  (0.40) |
| Average number of Glaucoma patients | Mean | 0.29 (0.45) | 0.19  (0.39) |
| SES | Mean | -0.24  (1.29) | -0.23  (1.20) |
| *Volume* | |  |  |
| Number of DRGs per patient journey of cataract care | Mean | 1.47  (0.73) | 1.40  (0.61) |
| Number of cataract operations per patient journey | Mean | 0.67  (0.67) | 0.86  (0.79) |
| >=2 cataract operations per patient journey | % | 11.1 | 24.63 |
| *Price* | |  |  |
| Price DRG for standard cataract surgery | Mean | 1207.11  (72.23) | 1091.45  (127.16) |
| Price DRG for complex cataract surgery | Mean | 1844.82  (495.14) | 1361.35  (105.61) |
| *Total costs* | |  |  |
| Total costs for cataract care – conservative | Mean | 173.63  (131.14) | 119.05  (72.79) |
| Total costs for patients with 1 cataract | Mean | 1413.34  (370.80) | 1149.67  (173.48) |
| Total costs for patients with 2 cataract | Mean | 2734.85  (623.77) | 2262.29  (284.93) |
| Standard deviations in parentheses | | | |

Table S2. Descriptive statistics: provider characteristics, type of treatments, patient characteristics, volume, price and total costs (2013)

|  | | **ITCs** | **GHs** |
| --- | --- | --- | --- |
| *Provider characteristics* | |  |  |
| Total number of providers | N | 29 | 62 |
| Number of patients | N | 10097 | 24656 |
|  | % | 19.3% | 47.0% |
| *Type of treatment* | |  |  |
| Standard cataract surgery | % | 56.37 | 55.34 |
| Complex cataract surgery | % | 5.85 | 3.77 |
| No surgery | % | 37.77 | 40.89 |
| *Patient characteristics* | |  |  |
| Average Age | Mean | 72.02 (9.72) | 72.98 (10.12) |
| <18 years | % | 0.03 | 0.20 |
| >85 years | % | 8.07 | 9.64 |
| Men | % | 39.83 | 42.34 |
| Average number of chronic conditions | Mean | 2.11 (1.65) | 2.28 (1.70) |
| Average number of Diabetes I patients | Mean | 0.04 (0.20) | 0.09 (0.28) |
| Average number of Diabetes II patients | Mean | 0.13 (0.34) | 0.20 (0.40) |
| Average number of Glaucoma patients | Mean | 0.30 (0.46) | 0.12 (0.32) |
| SES | Mean | -0.07  (1.24) | -0.29  (1.16) |
| *Volume* | |  |  |
| Number of DRGs per patient journey of cataract care | Mean |  |  |
| Number of cataract operations per patient journey | Mean | 0.90 (0.81) | 0.80 (0.76) |
| >=2 cataract operations per patient journey | % | 27.6 | 20.97 |
| *Price* | |  |  |
| Price DRG for standard cataract surgery | Mean | 1147.41 (45.28) | 1231.10 (178.03) |
| Price DRG for complex cataract surgery | Mean | 1316.49 (49.41) | 1363.57 (331.41) |
| *Total costs* | |  |  |
| Total costs for cataract care – conservative | Mean | 138.01 (74.83) | 119.45  (81.28) |
| Total costs for patients with 1 cataract operation | Mean | 1195.39 (102.62) | 1294.04  (219.36) |
| Total costs for patients with 2 cataract operations | Mean | 2350.67 (145.71) | 2487.15 (386.54) |
| Standard deviations in parentheses | | | |

Table S3. Descriptive statistics: provider characteristics, type of treatments, patient characteristics, volume, price and total costs (2014)

|  | | **ITCs** | **GHs** |
| --- | --- | --- | --- |
| *Provider characteristics* | |  |  |
| Total number of providers | N | 27 | 59 |
| Number of patients | N | 11072 | 21875 |
|  | % | 22.5 | 44.4 |
| *Type of treatment* | |  |  |
| Standard cataract surgery | % | 55.21 | 55.50 |
| Complex cataract surgery | % | 6.42 | 4.22 |
| No surgery | % | 38.35 | 40.27 |
| *Patient characteristics* | |  |  |
| Average Age | Mean | 72.20  (9.85) | 72.98  (10.02) |
| <18 years | % | 0.08 | 0.15 |
| >85 years | % | 8.03 | 9.66 |
| Men | % | 39.51 | 41.81 |
| Average number of chronic conditions | Mean | 2.12  (1.64) | 2.24  (1.70) |
| Average number of Diabetes I patients | Mean | 0.05 (0.22) | 0.08  (0.28) |
| Average number of Diabetes II patients | Mean | 0.13  (0.34) | 0.19 (0.39) |
| Average number of Glaucoma patients | Mean | 0.29 (0.45) | 0.12 (0.33) |
| SES | Mean | -0.06  (1.20) | -0.31 (1.18) |
| *Volume* | |  |  |
| Number of DRGs per patient journey of cataract care | Mean | 1.40 (0.58) | 1.34  (0.57) |
| Number of cataract operations per patient journey | Mean | 0.86 (0.78) | 0.78  (0.74) |
| >=2 cataract operations per patient journey | % | 24.73 | 18.62 |
| *Price* | |  |  |
| Price DRG for standard cataract surgery | Mean | 1072.08 (73.01) | 1164.57 (156.39) |
| Price DRG for complex cataract surgery | Mean | 1284.27 (83.87) | 1451.95 (204.28) |
| *Total costs* | |  |  |
| Total costs for cataract care – conservative | Mean | 129.05  (67.51) | 123.76 (71.14) |
| Total costs for patients with 1 cataract operation | Mean | 1128.21  (124.68) | 1216.40 (189.48) |
| Total costs for patients with 2 cataract operations | Mean | 2193.03 (180.31) | 2387.61 (341.06) |
| Standard deviations in parentheses | | | |

Table S4. PROM questions - in Dutch and with our own English translation

| Hoe gaat het volgende bij u: goed dichtbij kunnen zien?  *How would you assess the following: seeing well close by?* | | | | | | | |
| --- | --- | --- | --- | --- | --- | --- | --- |
| Hoe gaat het volgende bij u: samenwerking tussen beide ogen?  *How would you assess the following: coordination between both eyes?* | | | | | | | |
| Hoe gaat het volgende bij u: gevoel van onafhankelijkheid?  *How would you assess the following: sense of independence?* | | | | | | | |
| Hoe gaat het volgende bij u: goed veraf kunnen zien?  *How would you assess the following: seeing well from far away?* | | | | | | | |
| Hoe gaat het volgende bij u: doen van de normale bezigheden?  *How would you assess the following: doing normal activities?* | | | | | | | |
| Hoe gaat het volgende bij u: geen last hebben van felle lichten?  *How would you assess the following: bright lights bothering you?* | | | | | | | |
| Hoe gaat het volgende bij u: mogelijkheid tot deelname aan het verkeer?  *How would you asses the following: possibility to participate in traffic?* | | | | | | | |
| Hoe gaat het volgende bij u: geen dingen dubbel zien?  *How would you assess the following: not seeing things in double vision?* | | | | | | | |
| Hoe gaat het volgende bij u: goed op middelgrote afstanden kunnen zien?  *How would you assess the following: good vision at medium distances?* | | | | | | | |
| Hoe gaat het volgende bij u: helder en kleurrijk zien van dingen?  *How would you assess the following: seeing the colors clearly?* | | | | | | | |
| Hoe gaat het volgende bij u: goed erg dichtbij kunnen zien?  *How would you assess the following: seeing well from very close by?* | | | | | | | |
| Hoe gaat het volgende bij u: geen dingen wazig zien?  *How would you assess the following: not seeing things blurred?* | | | | | | | |
|  | | | | | | | |
| *Options for answering2013* | | | | | | | |
| Veel slechter dan verwacht  *Much worse than expected* | Slechter dan verwacht  *Worse than expected* | | Zoals verwacht *As expected* | | Beter dan verwacht  *Better than expected* | | Veel beter dan verwacht  *Much better than expected* |
| *Options for answering2014* | | |  | |  | |  |
| Nu veel slechter dan voor de operatie  *Now much worse than expected* | | Geen verschil  *No difference* | | Nu beter dan voor de operatie  *Now better than expected* | | Nu veel beter dan voor de operatie  *Now much better than expected* | |
